# Prediction of transportation index for urban patterns in small and medium-sized Indian cities using hybrid RidgeGAN model

**DOI:** 10.1038/s41598-023-49343-3

**Published:** 2023-12-10

**Authors:** Rahisha Thottolil, Uttam Kumar, Tanujit Chakraborty

**Affiliations:** 1 Spatial Computing Laboratory, Center for Data Sciences, International Institute of Information Technology, Bangalore, 560100 India; 2 Department of Science and Engineering, Sorbonne University Abu Dhabi, Abu Dhabi, United Arab Emirates; 3 School of Business, Woxsen University, Hyderabad, Telangana India; 4 Sorbonne Center for Artificial Intelligence, Sorbonne Université, Paris, France

**Keywords:** Sustainability, Computational science, Information technology, Scientific data, Geophysics

## Abstract

The rapid urbanization trend in most developing countries including India is creating a plethora of civic concerns such as loss of green space, degradation of environmental health, scarcity of clean water, rise in air pollution, and exacerbated traffic congestion resulting in significant delays in vehicular transportation. To address the intricate nature of transportation issues, many researchers and planners have analyzed the complexities of urban and regional road systems using transportation models by employing transportation indices such as road length, network density, accessibility, and connectivity metrics. This study addresses the complexities of predicting road network density for small and medium-sized Indian cities that come under the Integrated Development of Small and Medium Towns (IDSMT) project at a national level. A hybrid framework based on Kernel Ridge Regression (KRR) and the CityGAN model is introduced to predict network density using spatial indicators of human settlements. The major goal of this study is to generate hyper-realistic urban patterns of small and medium-sized Indian cities using an unsupervised CityGAN model and to study the causal relationship between human settlement indices (HSIs) and transportation index (network density) using supervised KRR for the real cities. The synthetic urban universes mimic Indian urban patterns and evaluating their landscape structures through the settlement indices can aid in comprehending urban landscape, thereby enhancing sustainable urban planning. We analyzed 503 real cities to find the actual relationship between the urban settlements and their road density. The nonlinear KRR model may help urban planners in deriving the network density for GAN-generated futuristic urban patterns through the settlement indicators. The proposed hybrid process, termed as RidgeGAN model, can gauge the sustainability of urban sprawl tied to infrastructure and transportation systems in sprawling cities. Analysis results clearly demonstrate the utility of RidgeGAN in predicting network density for different kinds of human settlements, particularly for small and medium Indian cities. By predicting future urban patterns, this study can help in the creation of more livable and sustainable areas, particularly by improving transportation infrastructure in developing cities.

## Introduction

Mapping urban land use dynamics has been valuable research in urban studies over several decades. The advancement in remote sensing technology makes it possible to track the spatiotemporal changes in urban landscape structures with relatively high accuracy and on a required scale^1^. The spatial distribution of land use activities (residential, commercial, industrial, etc.) including the transportation system is important for understanding the current urban centers and for planning future city development^2^. Land use and land cover maps become a starting point for modeling urban patterns and they can infer the future urban growth and the direction of land expansion of cities to inform urban planners and government policymakers towards sustainable urban planning^3–5^. While urban areas continue to experience rapid growth, they pose new challenges to the nation, especially for developing and underdeveloped countries^6^. Hence, urban growth prediction models and related studies have become a hot topic that has been extensively and deeply investigated. The existing urban growth models^7–10^ include the driving factors which affect urban expansion. These factors influencing urban expansion include population growth, economic development, urbanization, transportation infrastructure, topography, and land use regulations^11^. However, in developing and underdeveloped nations, where urban expansion is more likely to occur, data on driving forces are hard to obtain and are often expensive to collect.

According to the UN report^12^, India is the most populous country in the world. From 1901 to 2011, the country’s urban population expanded by around 14 times^13^. Although largely unequal, its increase is not skewed and is not limited to one region across the nation. The skyrocketing living costs in metropolitan areas and increasing house rents discourage enterprises from investing in major cities. Therefore, it is essential to assess the settlement pattern and infrastructure facilities of small and medium towns as an alternate option to larger metropolitan cities^14^. These towns sometimes called the “next billion” markets, will be important in propelling the expansion of the national economy in India^15^. Further to the intricacy of urban settlement, more basic infrastructure and facilities are required for these regions. Various reports in recent years have estimated a massive demand for funding urban infrastructures in developing countries. The World Bank estimates that nearly 70,000 billion (INR) of investment in urban India will be required to meet the growing population demands in the next 15 years until 2036 (in 2020 prices)^16^. For example, the Indian Government introduced a scheme called the Integrated Development of Small and Medium Towns (IDSMT) that aims to encourage the planned and sustainable growth of the nation’s small and medium-sized towns or cities^15^. The Ministry of Urban Development, Government of India introduced this scheme in 2005, and it offers financial and technical assistance to local governments in order to help them develop their towns’ infrastructure and fundamental services. This scheme focuses on the expansion of small and medium-sized towns (the population size is up to 500 K), which may act as growth hubs for the nearby rural regions, in order to promote inclusive growth and balanced regional development. By providing funding for the construction of fundamental utilities including water supply, sanitation, solid waste management, and urban transportation, the program seeks to solve the infrastructure deficit and service shortages in these communities. The overall goal of the IDSMT program is to support sustainable urban growth and raise the standard of living in India’s small and medium-sized towns. As a result, it is important to examine the small and medium towns (Tier 3 and above cities with populations up to 500 K) in India. Adopting cutting-edge technologies can have a significant impact on enhancing Government’s effectiveness in improving planning and decision-making, problem-solving, accelerating development, and deployment^17^.

Urban planning is a complex field that faces numerous challenges as cities continue to grow and evolve. Some of the key challenges include rapid urbanization, housing shortages and affordability, transportation and traffic problems, environmental sustainability, and urban infrastructure deficits, etc. Understanding and predicting traffic conditions is a complex task and often involves a combination of quantitative data analysis, modeling, and expert judgment. Prediction models are essential for understanding the transportation infrastructure’s capacity, identifying potential bottlenecks, and planning for future growth and development. It is also useful when planning new infrastructure projects. By understanding future transportation demands, city planners can determine the need for new roads, bridges, public transportation hubs, and bike lanes, facilitating better infrastructure development for future transportation needs. The field of transportation modeling is continually evolving, with advancements in machine learning and data analytics contributing to more accurate and sophisticated prediction models.

Another key issue is the rapid evolution of new cities in developing countries. Addressing these challenges requires learning from the experiences of established cities and adapting best practices to the specific context of each new city is essential for successful development. Recent developments in machine learning and deep learning have become handy tools for urban planners and geoscience practitioners. Deep learning (DL) has gained significant attention in recent years for its ability to model complex patterns and make accurate predictions and it has the potential to address several urban planning challenges as well. Deep learning models such as generative adversarial networks (GAN) can approximate complex, high-dimensional probability distributions^18^. GANs have achieved numerous state-of-the-art breakthroughs in the fields of computer vision^19^, natural language processing^20^, and more recently in urban science and geospatial domain^21,22^. Several GAN-based models have been proposed to simulate hyper-realistic urban land use maps and generate synthetic urban universe without considering the driving factors, for example, CityGAN^23^ and MetroGAN^24^. Among these, CityGAN^23^ simulates urban patterns using global urban land-use inventory and builds an “urban universe” to reproduce the complex spatial patterns observed in global cities. An extension to CityGAN by incorporating geographical knowledge is called Metropolitan GAN (MetroGAN)^24^ which learns hierarchical features for urban pattern simulation. Another deep learning method, namely U-Net^25^, is also applied to generate future urban cities using water bodies, digital elevation models, and nighttime lights as inputs^22^. The application of previous GAN-based urban simulation models was limited to the generation of urban patterns. Applications of deep learning models in transportation sectors are the prediction of traffic conditions^26^ and transportation safety planning with high-resolution transportation and land use data^27^. Recently, authors^28^ discussed the implementation of a “Smart Trash Management and Categorization System” to address the challenges of solid waste management, disposal, and recycling using a combination of the Internet of Things (IoT) and deep learning techniques. By analyzing real-time data from IoT sensors, deep learning models can optimize the use of resources and detect anomalies or potential issues. By harnessing the power of deep learning and combining it with traditional urban planning methodologies, cities can make more data-driven, efficient, and sustainable decisions to tackle the complex challenges they face. There are limited works on quantifying the urban pattern of GAN-generated images and predicting the transportation index (representing urban infrastructure) for the new urban regions. Thus, quantification and modeling of urban patterns of GAN-simulated cities remain an unattempted problem. The structure of an urban landscape emerges from the characteristics of the individual elements of an ecosystem and their spatial configuration^29–32^.

Even though hundreds of settlement indices are available to analyze urban landscapes, different indices capture different aspects of landscapes. Human Settlement Indices (HSIs), e.g., Class Area (CA), Number of Patches (NP), Largest Patch Index (LPI), Clumpiness Index (CLUMPY), Aggregation Index (AI), and Normalized Landscape Shape Index (NLSI)^33–35^ provide some concrete information about the landscape structures and therefore contributes in the prediction of Transportation Index (TI). The above-mentioned six indices are most relevant and meaningful for the specific characteristics of the urban landscape and are widely recognized and standardized within the urban research field. The indices are represented as HSIs in this research because we analyze human settlement patterns in an urban area. Transportation index refers to a metric or indicator used to assess and measure the efficiency, accessibility, and sustainability of road transportation systems within a city or urban area. It is a way to quantify how well a city’s transportation network is meeting the needs of its residents and visitors. Understanding road conditions in a city helps to address traffic congestion by identifying areas with low density and improving its infrastructure. Road network density is considered as TI in this research, which refers to the extent and complexity of the road infrastructure within the urban area in terms of its density^36^. It is a measure of how well the roads are interconnected and distributed throughout the city. It provides an indication of how much road infrastructure exists per unit of land area^37^. Road network density is an important aspect of urban planning and transportation management as it directly affects traffic flow, accessibility, and overall mobility within the city. Higher road density means more roads are present in a given area, which can lead to better accessibility. By considering a single transportation index, urban planners and policymakers can gain insights into the strengths and weaknesses of the transportation system. This, in turn, helps them make informed decisions about investments, infrastructure improvements, and policy changes to create a more efficient, sustainable, and inclusive urban transportation network.

This paper makes an attempt to answer the following challenging questions:

- How to generate an urban universe for India based on spatial patterns via learning urban patterns?
- Is there a relationship between HSI and TI in small and medium cities in India?
- How to predict (forecast) TI for synthetic urban cities generated by CityGAN for developing countries like India?

To achieve the above-mentioned goals, we deployed the CityGAN model that can generate realistic urban patterns to capture the great diversity of urban forms across small and medium-sized Indian cities. CityGAN is the first step towards a flexible urban pattern simulator for more accurate projections on urbanization in regions where local data such as the location of services, population density, and other socio-economic factors are unavailable and difficult to obtain. In the case of a developing country like India, data on these spatial co-variates are difficult and expensive to compile and are often not available, where urban growth is more likely to occur. Hence the CityGAN was selected in our research based on the limited number of available urban maps. It is computationally efficient for generating Indian urban maps without considering feature datasets compared to other existing GAN models e.g., MetroGAN, and UNet.

To generate small and medium-sized Indian cities’ urban maps with CityGAN, we collected the World Settlement Footprints (WSF 2019) maps which are publicly available and the best representations of urban patterns as input features^38,39^. Then, we build a city image database of 503 small and medium Indian cities whose populations range between 20 and 500 K. Each city image represents $10.5 \times 10.5$ km covering the urban center and surrounding regions. We also explored existing spatial and statistical measures to evaluate the performance of CityGAN in Indian cities due to the complex nature of individual cities with varying structural and hierarchical properties^23^. Furthermore, we used the Chatterjee Correlation Coefficient and Kernel Ridge Regression to establish the relationship between HSIs and network density and to build a prediction model for TI (particularly, road network density) using HSI based on real Indian cities. Then, we propose a hybrid model (namely, RidgeGAN) to predict the transportation index for simulated urban patterns. Figure 3 depicts the overall methodological framework. In RidgeGAN, a supervised learning model (KRR) builds a relationship between the human settlement patterns and the characteristics of the urban road transportation system and implements this to predict TI for the GAN-simulated urban universe for India. This combined approach can be further used by urban planners to visualize and evaluate different urban development scenarios and optimize city layouts for specific objectives, such as planning road networks and minimizing traffic congestion among many others. By combining the creativity of GAN-generated cities with the data-centric KRR model, we can predict TI for urban patterns and can achieve more innovative and sustainable urban planning in the case of road networks. Other applications, range from understanding urban land patterns to predicting relevant urban infrastructure facilities to guiding policymakers toward a better and more inclusive planning process. City planners can use the simulated urban maps to explore different scenarios and understand the potential impacts of various development strategies, zoning regulations, and transportation infrastructure planning.

## Background and related work

### Applications of GANs in geospatial field

Deep learning has reached a significant milestone in geospatial research, computer vision, and other cutting-edge technologies^40^. GAN^18^, an essential subfield of unsupervised deep learning, has opened a new vista for geoscience research in recent years. GANs are utilized to generate data that is close to a given training set which can be images, texts, and tabular data^41^. Geoscientists and urban planners have adopted this new deep learning methodology for handling geophysical and remote sensing data. In remote sensing, MARTA GANs were proposed for producing fake satellite images of urban environments^42^. It consists of a discriminator network that receives both real and synthetic images as input and predicts whether each image is genuine or synthetic, as well as a generation network that uses random noise as input to create synthetic images. Further, Spatial Generative Adversarial Networks (SpaGANs)^43^ were introduced for synthesizing textures by incorporating spatial information (such as the position and orientation of the texture) into the generator and discriminator networks. A comprehensive review of GANs demonstrates promising performance in the built environment, from processing large-scale urban mobility data and remote sensing images at the regional level to performance analysis and design generation at the building level^22^.

As mentioned before, GANs were also applied to modeling global urban patterns. For example, CityGANs^23^, conditional GANs^44^, and MetroGANs^24^ were built to generate urban land patterns by training the generator networks to generate synthetic images of urban areas that closely resemble real urban areas and it will be useful for analyzing urban human settlement data from space-based sensors. For more accurate urbanization parameters or spatial indices estimates in locations where local data is unavailable or impossible to collect, these models are very effective in simulating urban land use patterns. GANs are used to model hyper-realistic settlement patterns since they do not make any assumptions about the data distribution and can generate real-like samples from the latent space in a straightforward manner. This unique property lends GANs to a variety of geospatial applications, including image synthesis, image attribute editing, image translation, domain adaptation, and other computing fields^45^.

### Transportation and urban landscape structures

**Figure 1:**
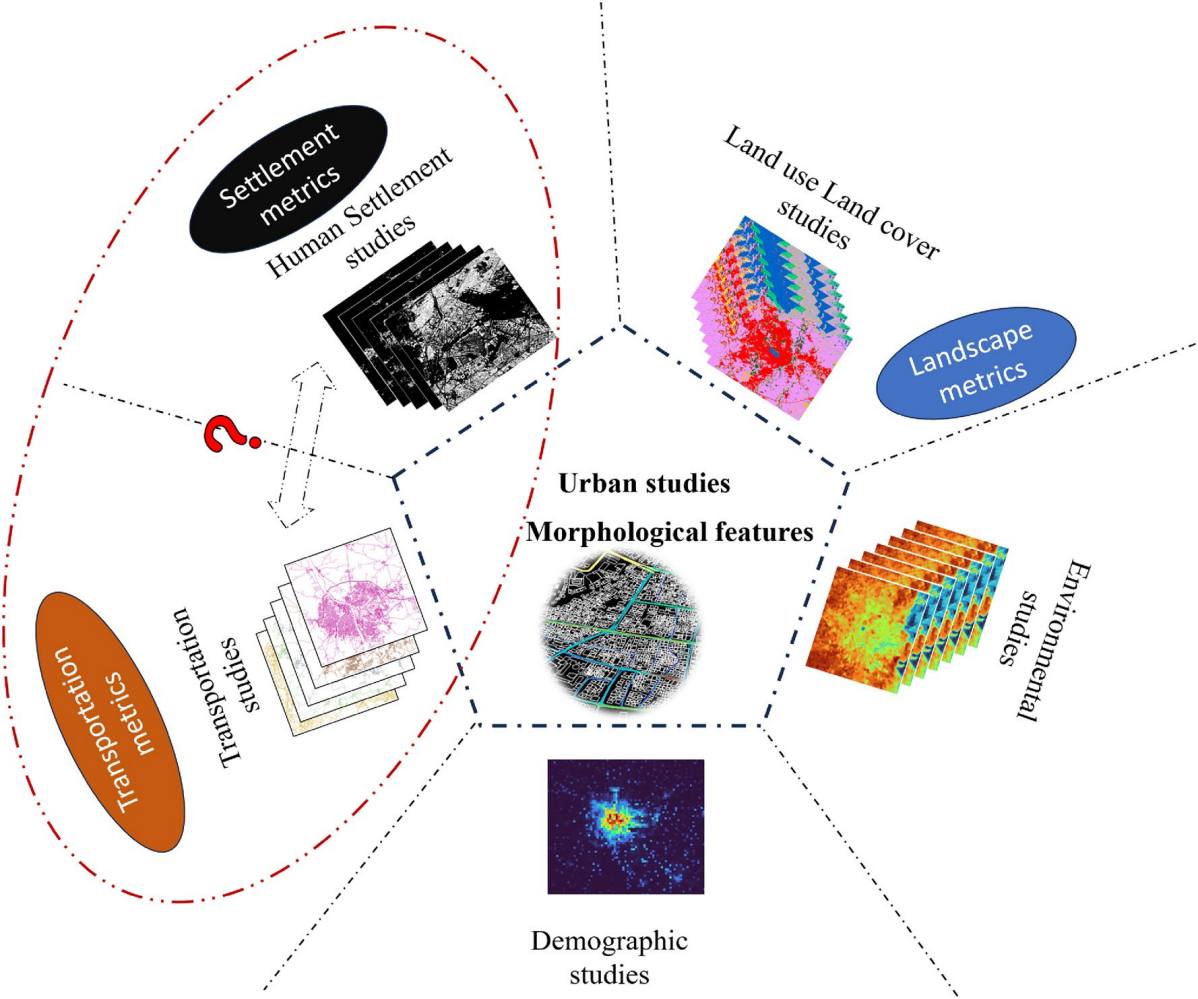
An overview of various urban metrics used in different types of urban studies. Various urban maps were generated by utilizing QGIS version 3.30 software^50^ and an overview of the entire figure was created using Microsoft PowerPoint (URL: https://www.microsoft.com/en/microsoft-365/powerpoint).

The spatial structure of a city is extremely complex and is constantly evolving. Delineating homogeneous/heterogeneous urban features, quantifying them, and analyzing their diversity and spatial organization are necessary to assess their structures and spatial patterns. Due to this, urban researchers utilized many metrics to analyze the characteristics of urban features related to shape, patterns, and area. Examples of commonly used urban metrics are human settlement indices, landscape metrics, transportation indices, and morphological features which are interconnected and influence each other in various ways. Figure 1 shows a new pictorial representation of various urban fields and corresponding metrics in urban studies. Urban morphology refers to the physical layout, structure, and design of settlements, particularly cities and towns. It involves studying the spatial arrangement of buildings, streets, open spaces, infrastructure, and other elements that make up the built environment within urban areas. Urban morphology features provide insights into how settlements are organized, how they have evolved over time, and how they function in terms of land use, transportation, and social interactions^24^. Generally, human settlement indices are measurements used to quantify aspects of human habitation patterns. These can include settlement density, settlement area, urban sprawl, land use patches, and more^38^. These indices provide insights into how people are distributed across a landscape and how urbanization is occurring. Whereas, landscape metrics are quantitative measures used to describe the spatial structure and pattern of urban landscapes^33^. Landscape metrics were originally introduced in ecological studies that reflected social, cultural, and ecological richness and heterogeneity^46^. Also, progressive and well-functioning urban planning departments can use spatial indicators to regularly monitor urban development and, when necessary, propose regulatory or public investment action^47^. These indicators can also evaluate the geometrical characteristics of ecological processes and landscape elements, as well as their relative locations and distribution^48^. The effect of landscape metrics on spatial patterns was studied to quantify landscape structures and these metrics can statistically determine the outcome^34^. Spatial patterns of urban growth and landscape metrics were studied for various cities in India^33,49^. Urban metrics provide valuable information about urban sprawl, fragmentation, and the overall spatial organization of the urban landscape. Urban landscape metrics are correlated with human settlement indices to assess various aspects of urban settlement patterns.

The transportation index referred to evaluates the transportation infrastructure and accessibility in urban areas, for example, road network density. A higher transportation index value indicates a developed road network and ease of movement within the urban area. They are closely linked to urban planning and can impact the development and expansion of human settlements. It is important to note that the relationships between these metrics are context-dependent and may vary across different cities and regions. Understanding the interaction between transportation infrastructures and urban pattern areas is always critical for driving smooth urban services. The investigation into the development of mathematical models for studying the relationship between transportation metrics and urban land use began in the early 1960s and technological advancements brought us to an era of integrated land use transportation modeling^51^. Several road network models have been developed to solve transportation problems. Most of the existing transportation models for prediction now in use are based on simple linear regression models. An inverse relationship between urban growth and transportation was found for the Middle East regions^52^. Their analysis suggested that urban population growth has increased urban trips and increased travel demand due to transportation infrastructure.

In a recent study, the link between population and the characteristics of the road network in the Lebanese Republic was investigated using a multivariate regression model to estimate the population count based on various data sources and statistical modeling techniques^53^. However, linear regression models have the drawback of excluding all variables that are not linearly connected, and multicollinear variables adversely affect the model. Besides, it is proved that the relationship between transportation features and their influencing factors is not always linear in nature. Predicting the transportation index is an important step towards minimizing traffic congestion and providing critical information to individual travelers as well as various Government sectors to plan the city in a sustainable way. A support vector regression (SVR) approach was used to predict traffic flow from California highways using different types of kernels^54^. A road network density (one of the transportation indices) prediction model was proposed using highway capacity, and turning probabilities manual methods were used to determine the shortest cycling time in metropolitan areas^55^. The model could aid in vehicle distribution and congestion relief in urban areas. Further, the concept of graph theory was used to analyze the topology of road networks in an Indian city to better understand the connectivity and coverage of the existing road transportation system^37^. Their findings indicate that there is a strong relationship between road connectivity and coverage and that improving the road network is essential for a reliable and safe road transportation system. To accurately estimate the connectivity index, the paper^37^ proposed a model based on the relationship between the Eta index and Network Density (ND), Edge Graph Density (EGD) and Nodal Graph Density (NGD). As per the previous literature^52,53^, researchers have been theories the fact that the higher population density (settlement) might lead to increased demand for transportation infrastructure, affecting the transportation index. Conversely, transportation access can also impact where settlements are located and how they develop. But no empirical evidence was given to prove how well these two variables are related. To fill that gap, we establish the relationship between the HSIs and TI based on the selected 503 small and medium real Indian cities.

## Results

The scholarly literature on urban challenges primarily focuses on megacities and large urban centers, but there are a great number of small and medium-sized cities in developing countries that should be prioritized. There is a pressing need to address the challenges of developing transportation networks and settlement patterns in these cities. Previous literature on urban studies does not adequately address the transportation, unplanned city growth, and socioeconomic and environmental challenges of small and medium-sized towns^56^. In this study, we utilize global training WSF data across India, and we demonstrate a simple and unconstrained GAN model to generate realistic settlement patterns that encompass the diversity of urban forms. We are primarily interested in how small and medium-sized cities are simulated using unsupervised CityGANs. Subsequently, an effective data-driven hybrid model is developed to predict road network density for a given urban settlement pattern.

### Study area

**Figure 2:**
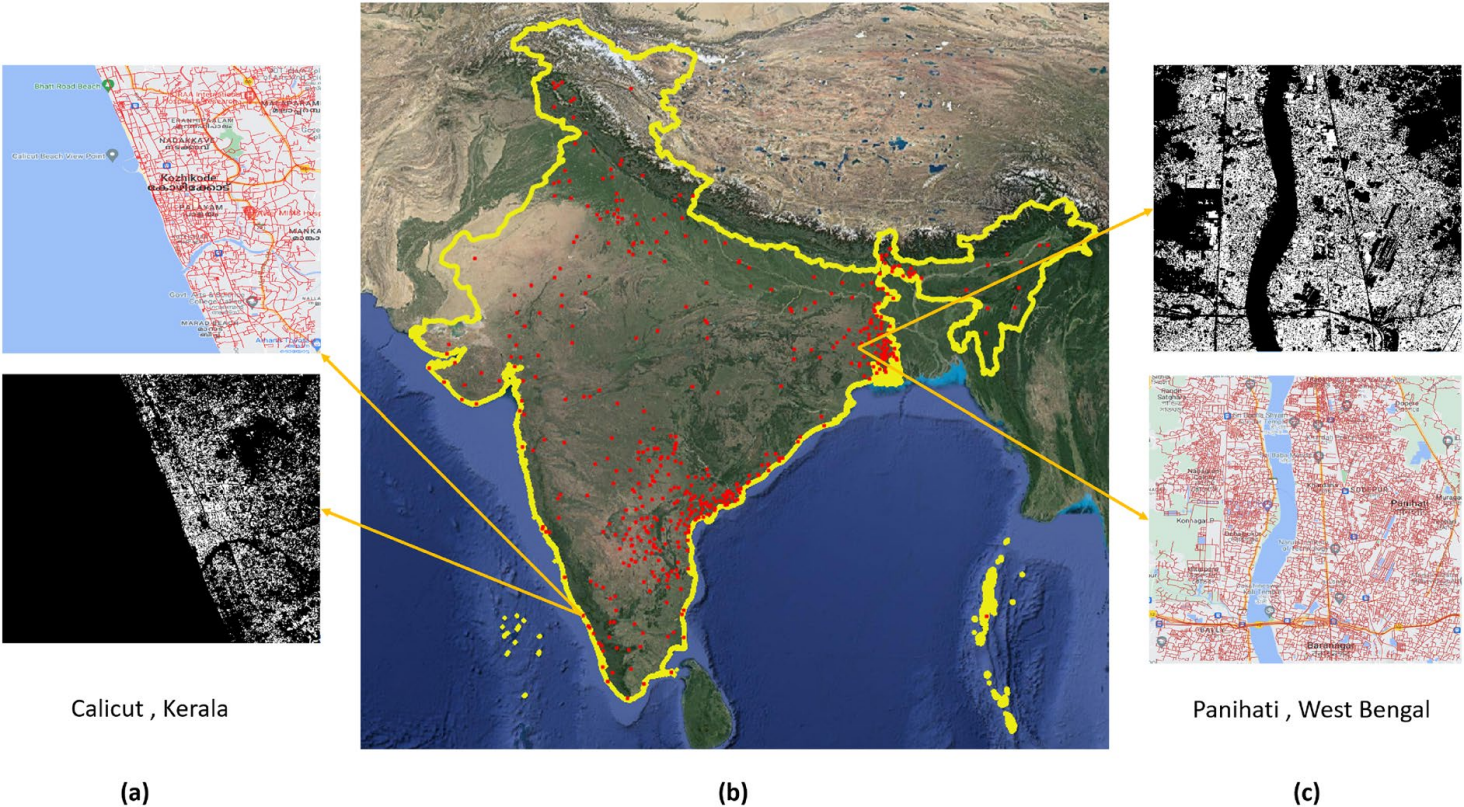
(**b**) Geographical distribution of 503 small and medium-sized Indian cities included in the study (red-colored square grids indicate the selected cities). Source: Maps data: Google, ©2015 India (URL: https://www.google.com/maps); (**a**) and (**c**) are examples of human settlement and transportation maps of two random cities, namely Calicut from the state of Kerala and Panihati from the state of West Bengal. Software used to generate (**a**) and (**b**) is QGIS 3.30^50^.

Our study focuses on small and medium-sized Indian cities (South Asia), one of the world’s fastest-urbanizing regions. The selection of the study area involved the identification of the geographical location and corresponding demographic data for which the population data from the World Cities database (https://simplemaps.com/data/world-cities) were used. Approximately, 503 cities out of 1600 were selected for our study where the population size ranges between 20 and 500 k. The geographical location of study areas marked in the map of India is depicted in Fig. 2b showing visualization of human settlement patterns and corresponding transportation networks (also see Fig. 2a,c).

### Data collection and preprocessing

Settlement footprints and transportation network datasets of all the selected cities were collated from various sources over 2019. Settlement maps were procured from the global inventory built-up database called WSF, published by the German Aerospace Center (DLR). These are binary maps (urban area pixels have a value of 1 and non-urban area pixels have a value of 0) derived from multi-temporal space-borne satellites namely Sentinel-1 and Sentinel-2 data that aided in estimating the human settlement pattern of an urban area ($10.5 \times 10.5$ km) with a resolution of around 10m/px. Open source GIS software (QGIS)^50^ was used to pre-process (rectify, project, and crop) the images and build a city database. To measure Human Settlement Indices (HSI), existing landscape metrics were selected and computed using Fragstat software^57^. It includes the packages to compute popular landscape metrics using spatial pattern analysis. We use six settlement indices: Total class area (CA), number of patches (NP), largest patch index (LPI), clumpiness index (CLUMPY), aggregation index (AI), and normalized landscape shape index (NLSI) to estimate the characteristics of human settlement^33,34^.

CA is a useful metric to depict the spatial extent of the settlements. It is a composition that specifies the extent of landscape that is made up of a specific class type (e.g., built-up area). The total class area, which is the sum of the areas (m$$^2 $$) of all the patches of the relevant patch type divided by 10,000 (converted to hectares) and CA $$> 0$$ indefinitely. NP in each landscape indicates the degree of fragmentation that counts the number of human settlements or urban patches. The higher the value of NP, the higher is the fragmentation with no limit. At the class level, LPI estimates the percentage of the total landscape area occupied by the largest patch as an indicator of dominance. LPI is calculated by dividing the area (m$$^2 $$) of the largest patch of the relevant patch type by the entire landscape area (m$$^2 $$) and multiplying the result by 100 (converted to a percentage), i.e., LPI is the percentage of the landscape comprised by the largest patch. It is a valuable index assessing fragmentation and is particularly useful when analyzing the distribution and the sizes of patches within a landscape. The high value of LPI indicates that settlements are fragmented into numerous smaller patches. Another human settlement metric CLUMPY deals with aggregation and disaggregation for adjacent settlements. It shows the frequency with which different pairs of patch types appear side by side. The value ranges from − 1 to 1; − 1 indicates maximally disaggregated patch type, 0 when the patch type is randomly distributed, and 1 when the patch type is maximally aggregated^57^. Another metric AI is calculated using an adjacency matrix, which indicates how frequently distinct pairs of patch types (including adjacencies between the same patch type) appear on the map side by side in the settlement map. Its values range from 0 to 100; AI values are less indicating maximum disaggregation and the high AI shows the maximally aggregated single and compact patch. Finally, NLSI provides a measure of class aggregation for which the values range from 0 to 1, where 0 means the landscape consists of a single square or maximally compact (i.e., almost square) patch. NLSI increases as the patch type becomes increasingly disaggregated and reaches 1 when the patch type is maximally disaggregated^33^.

**Figure 3:**
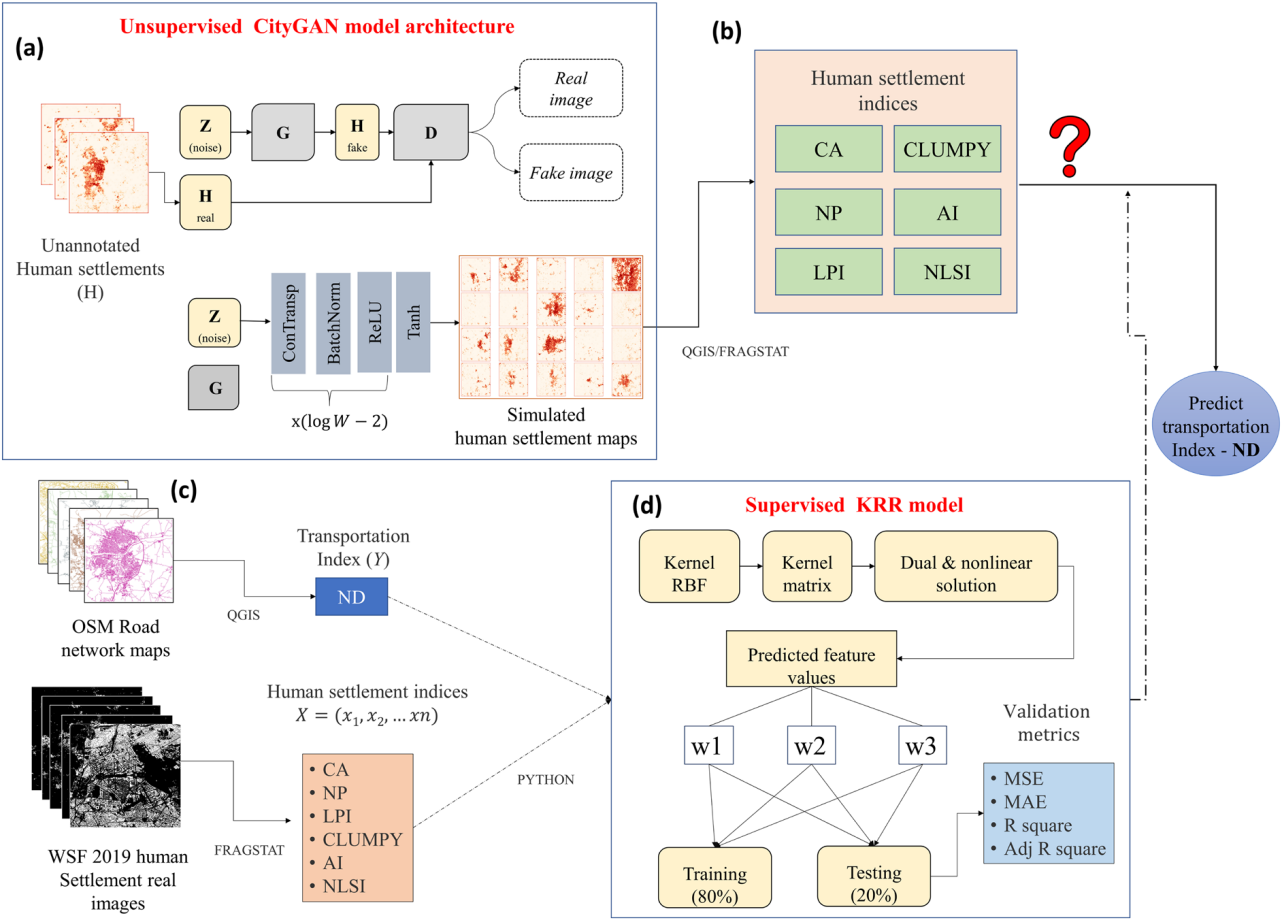
Prediction framework of the proposed hybrid RidgeGAN model: (**a**) implementing an unsupervised learning model (CityGAN) to generate small and medium-sized Indian cities; (**b**) landscape structures of generated cities are measured in terms of human settlement indices (HSI) using spatial landscape metrics; (**c**) characteristics of the road network and landscape structures of real cities are measured in terms of HSI and transportation index (TI); (**d**) assessing the relations between the settlement patterns and transportation system and building a supervised learning model to predict the transportation index for GAN-generated urban universe.

We have used OpenStreetMap (OSM) data to calculate the road network density for real small and medium-sized 503 Indian cities. While OSM data is a valuable and freely available resource for road network information, our current study focused on utilizing OSM road transportation datasets that align closely with the scope of our research objectives. To compute the Transportation Index (TI), a layer of the road network that has been topologically cleaned and converted into polylines is a prerequisite. The application software used here is integrated with QGIS 3.30^50^ for this purpose. Individual cities and their corresponding road networks were extracted to assess the spatial patterns of the road systems corresponding to the respective cities. As transportation measures need to be calculated in a metric system, a projected coordinate system was used. Instead of common WGS84-EPSG:4326, which uses degrees as a unit for distance, the Coordinate Reference System WGS84/UTM-EPSG was used here, to measure road length in meters. All categories of roads such as National highways, State highways, major roads, street roads, residential paths, footways, and service roads were included in this study. To measure the development of the urban road network, the network density of the respective cities was computed as follows:

$$\begin{aligned} \text {Network Density} \; (ND) = \frac{L}{A} = \frac{\text {Total length of the road network}}{\text {City area}}; \end{aligned}$$

where *L* is determined from road maps and it has been calculated using an open field calculator in QGIS software^50^. Network length specifies the total span of the road network and network density is measured according to the area occupied by road networks (city area), denoted as *A*, which refers to a geographical region characterized by a small and medium population. In real-world scenarios, the boundaries of a city area can vary based on administrative definitions. In our case, the city area is defined as approximately 10.5 km square grid from the city center, hence the city area is the same for all the cities approximately, 110.25 km$^{2}$. Figure 3 shows the detailed workflow of the proposed hybrid framework used in this study to predict the transportation index for any kind of urban pattern in Indian cities.

### Validation metrics

In this section, we estimate the Average Radial Profile (*ARP*) for real and generated urban patterns to assess the accuracy of CityGAN quantitatively. While CityGAN models can simulate hyper-realistic urban patterns, it is essential to ensure that the generated cities align with real-world urban patterns. Although, generated images were not able to be distinguished from real images by human visual inspection as a quantifiable measure; we utilized the existing *ARP* method, which is very commonly used in spatial summary statistics in urban forms to compare the polycentric nature of two cities, real and generated cities. An example of computing the *ARP* of a city is illustrated in Fig. 8 and using a peak search algorithm, we determined the polycentricity of actual and simulated scenes from the radial profiles. City centers were obtained from the World City database, which provides the geographical location of each city. Squared shape settlement maps of cities from WSF images were obtained by considering this city center and buffer operation in QGIS software^50^. *ARP* is denoted as *h*(*d*) or *x*(*d*) representing how much the settlement area changes as we go out from the city center. As indicated in Fig. 8b, we draw rings at a distance of *d* from the center and a width $\Delta$ of *d*. *ARP* can be computed by averaging the total amount of settlement area within the rings of width $\Delta d$ at a distance *d* from the city center. The region inside the ring is denoted as *R*(*d*) and each pixel inside the ring has some build-up area, that is the amount of urbanized area denoted as *h*(*u*, *v*), where (*u*, *v*) is a point inside the ring $h(u,v) \in R(d)$, with

1 $$\begin{aligned} \begin{aligned} R(d) \equiv \{ (u, v) \mid (u-u_0)^2+(v-v_0)^2>d^2 \; \; \text {and} \; \; (u-u_0)^2+(v-v_0)^2 \le (d+\Delta d)^2\}; \end{aligned} \end{aligned}$$

2 $$\begin{aligned} h(d)=\frac{1}{|R(d)|} \sum _{(u, v) \in R(d)} {h(u,v)}; \end{aligned}$$

where the city center is denoted as $(u_0,v_0)$ and *R*(*d*) can be defined as the collection of all the two-dimensional points included within the ring, |*R*(*d*)| indicates the size of set *R*(*d*), and *h*(*d*) is the average radial profile of a city. City centers were obtained from the World City Database, which provides the geographical location of each city. Squared shape settlement maps of cities from WSF images were obtained by considering this city center and buffer operation in QGIS software^50^.

There are several statistical measures that are used to assess the supervised regression models, for e.g., mean squared error (MSE), mean absolute error (MAE), R-squared ($R^2$), and adjusted R-squared (Adj $R^2$). MSE is the average squared difference between the predicted and actual values. It is a widely used metric that measures the quality of a regression model whereas MAE is the average absolute difference between the predicted and actual values. It is a robust metric that is less sensitive to outliers than MSE. R-squared measures the proportion of variance in the target variable that is explained by the model. It ranges from 0 to 1, with higher values indicating a higher correlation. adjusted R-squared is a modified version of R-squared that takes into account the number of predictors in the model. It penalizes the model for adding unnecessary variables and is a better measure of a model’s goodness of fit^58^.

### Generating human settlement area from WSF 2019

A dataset of real-time human settlement images was collected and pre-processed before training the GAN. We utilized squared-shaped settlement maps of cities from the WSF 2019 database. We clipped them representing 10.5 km $\times$ 10.5 km spatial extent and resized each image to 256 $\times$ 256 (43 m/px) for optimization purposes and to avoid overfitting. The final input dataset consists of 503 real images as binary maps and can be formulated as $H_i = {h_1,\ldots ,h_n}$, with $H_i\in R^{W X W}$ and $W=256$ and $n=503$. The element of $H_i$ is an urban binary map (1 and 0 represent urban and non-urban areas respectively). We chose 503 binary maps of Indian cities to align with the objectives of the Integrated Development of Small and Medium Towns (IDSMT) National Scheme. This selection ensures that the model is trained on a dataset that is both representative and manageable for effective training. The generator network is trained to generate synthetic urban settlement images by generating random noise and transforming it into an urban image whose distribution matches the real urban images. The discriminator network is trained to distinguish between real and synthetic binary images. CityGAN architecture contains a Generator and Discriminator, both are deepCNN with different weight vectors. In Generator, we first append Inverse CNN layers along with batch normalization, and Relu activation function, and add tan hyperbolic activation function. For Discriminator, we used a very similar architecture, with LeakyRelu, a nonlinear activation function instead of Relu, and simple CNN instead of inverse CNN. During parameter optimization, we use the Adam optimizer with a learning rate of 0.0002 and weight (decay rate) parameter Beta ranges from 0.5 to 0.999. We initialize the train loop with 25 sets of epochs. Both the G and D are trained with a loss function as discussed in the main manuscript.

**Figure 4:**
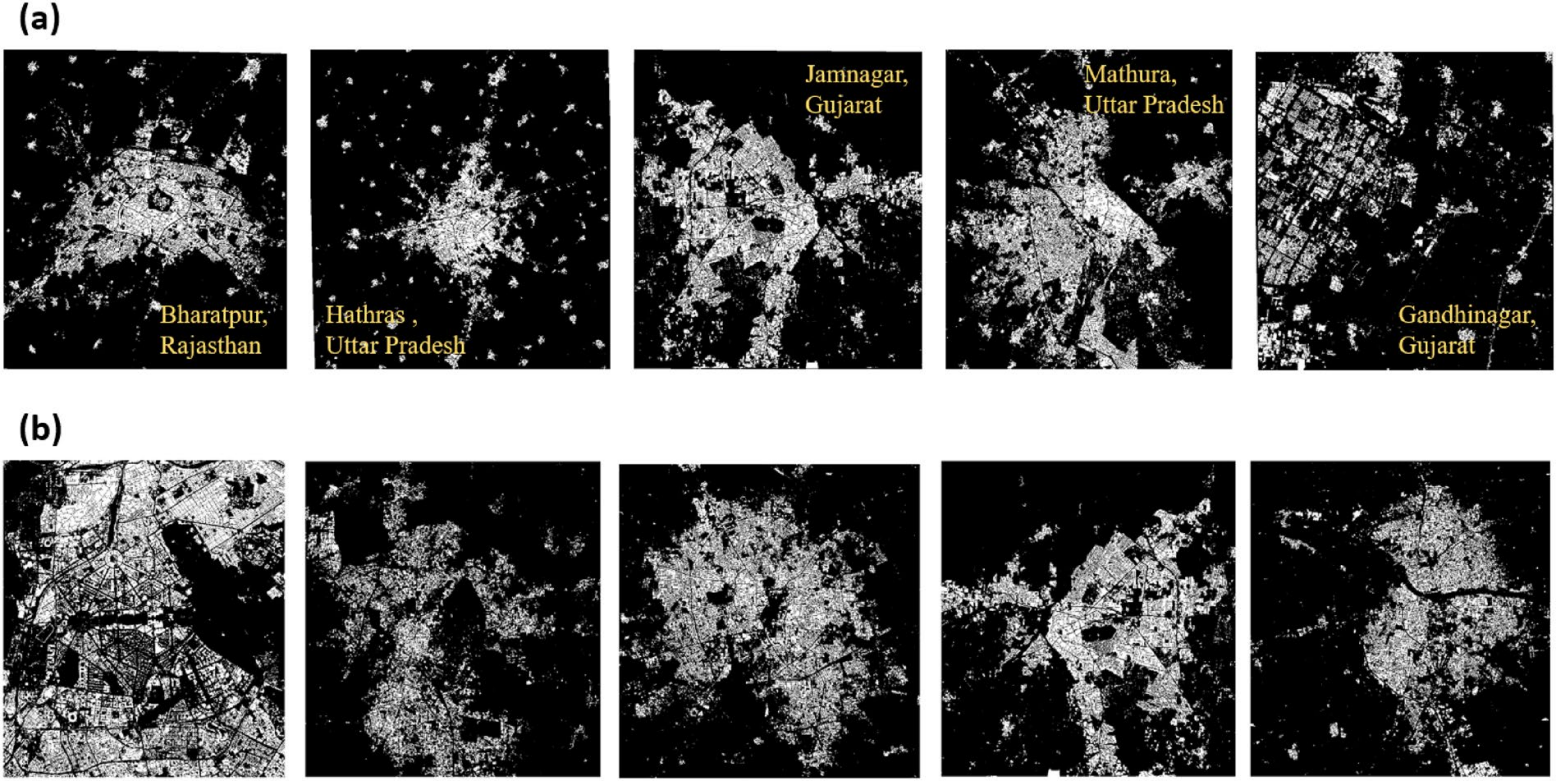
Comparison of real urban built land use maps (**a**) and synthetic maps (**b**) generated by a CityGAN. The pixel values in each case are in the range [0, 1], where 1 represents the portion of land occupied by buildings. Names of the cities are reported in (**a**) using yellow color text. The real and simulated images are loaded in QGIS version 3.30 software^50^.

Generator (*G*) takes a random noise vector $Z_{noise}$ as input, which deterministically changes (e.g., by passing it through successive deconvolutional layers if *G* is a deep CNN) to generate a sample fake human settlement image $H_{fake} = G(z)$. Then the Discriminator (*D*) accepts an input image *H* (which can be an actual human settlement image ($H_{real}$) from an empirical dataset or generated image ($H_{fake}$) synthetically by a generator and outputs the source probability that *H* is either sampled from the real distribution ($H_{real}$) or produced by generator ($H_{fake}$). Having trained a generator (*G*) (refer Fig. 3a), we generated synthetic Indian urban settlement patterns of 500 binary images using the CityGAN model. Despite the capability to generate thousands of synthetic images using the CityGAN model, we deliberately limited the output to only 500 images to balance the computational resources and the need for a representative dataset of small and medium-sized Indian settlement patterns. We found that the generated 500 binary images provide an effective representation of the diversity and characteristics of the Indian cities. Figure 4 illustrates randomly selected real cities (Fig. 4a) and simulated urban patterns (in Fig. 4b). On a visual inspection, simulations are practically indistinguishable from the actual urban patterns, with realistic densities and complexity of settlement patterns. Input images and generated images exhibit realistic concentration at the center and distribution of settlement in the surrounding regions. Various quantitative metrics as discussed earlier are used to evaluate the performance of the Indian CityGAN model^47^.

**Figure 5:**
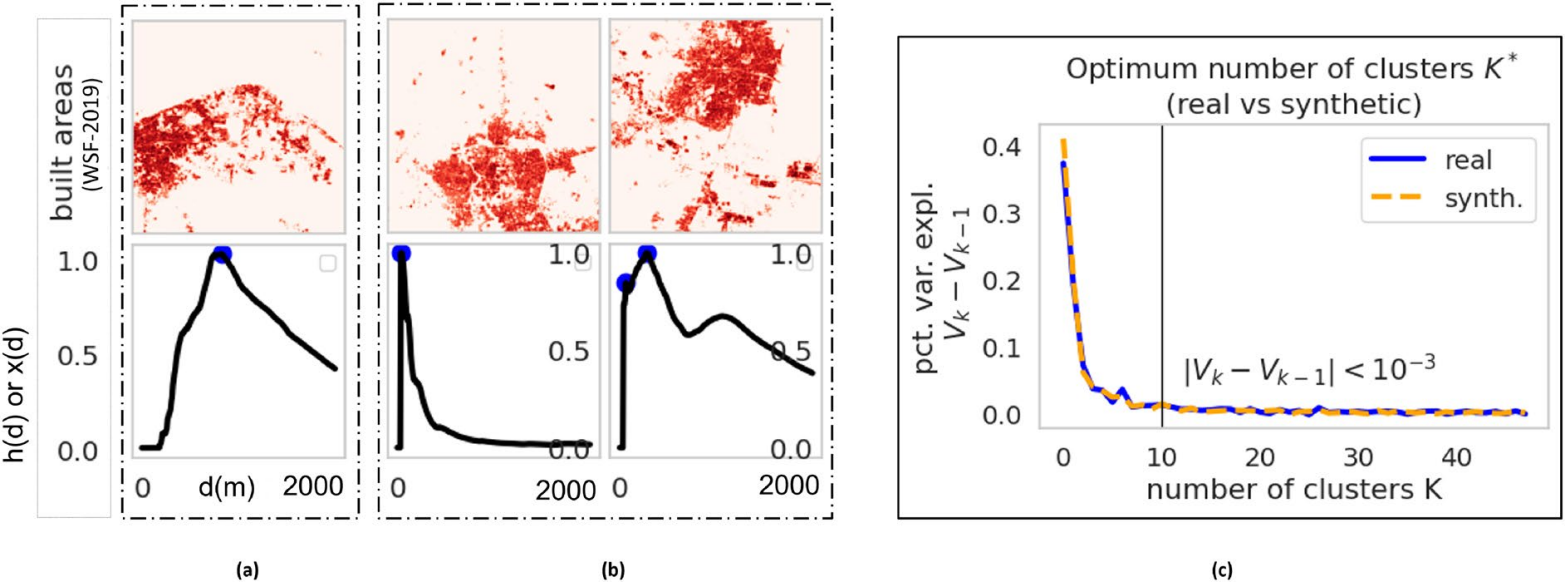
(**a**) Average radial profile map of Bhagalpur city; (**b**) human settlement maps of two example cities (top row) along with their average radial profiles (bottom row), where the blue dots represent the peaks found by the peak-search algorithm; (**c**) clustered radial profiles of real and synthetic settlement patterns for determining the number of clusters.

Among various spatial statistic measures, ARP^23^ is used to compare two cities, real and simulated urban patterns. We utilize Eq. (2) to compile the polycentric nature of real and generated images via the peak search algorithm illustrated in Fig. 8. The peak search algorithm finds points in univariate profiles whose value (peak height) is a fraction of the maximum height *h* and at a distance from the previously identified peak of at least $\delta$. We set an acceptable value of $h=80\%$ and $\delta = 430$ m via the cross-validation method. Graphical representations of average radial profiles and peaks (blue dots) from peak search algorithms for three randomly selected cities are illustrated in Fig. 5a,b.

**Figure 6:**
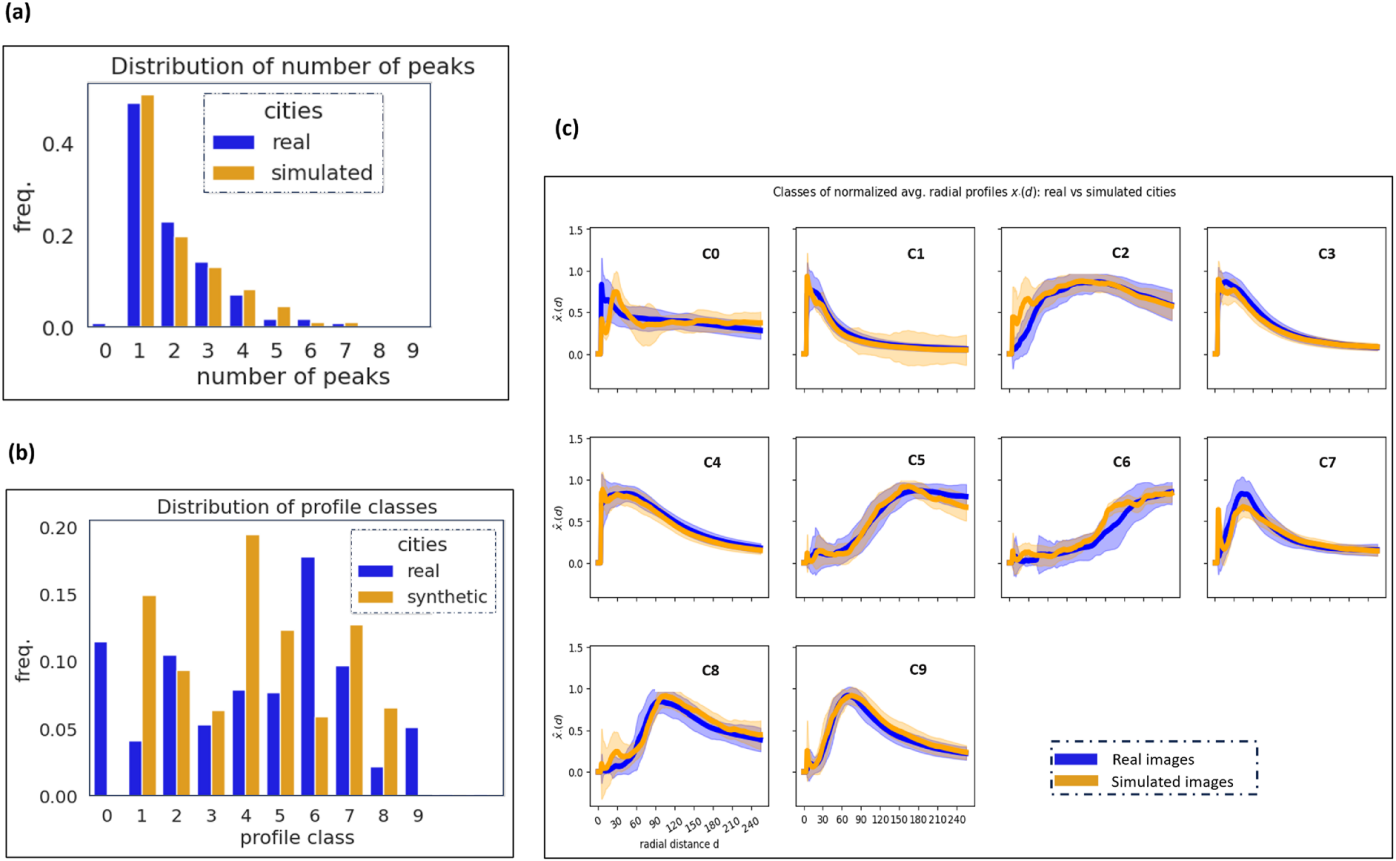
(**a**) Comparison of the distribution of the number of peaks of real and generated cities; (**b**) comparison of the distribution of average radial profile classes of real and generated cities; (**c**) the typical radial profiles for real and generated Indian cities (similar profile).

The distribution of the number of peaks for real and fake cities is compared (refer to Fig. 6a) and similarities between the distributions are found. Further, we cluster the radial profiles of real cities using the K-means algorithm^59,60^ and compare it to the typical profile of generated cities. Figure 6 presents a summary of our findings from the CityGAN model. Our analysis suggests that $K^*=10$ (refer Fig. 5c) gives the optimal number of clusters for both actual and synthetic scenes using a straightforward fraction of the sum-of-squares argument^60^. In Fig. 6c, the distribution of scenes by class is given and they have a similar shape and are more comparable. But in Fig. 6b, we find more disparities for classes 1 and 4 (monocentric) and class 6 (sprawled patterns). These discrepancies may result from a sampling technique that would have favored the abundance of mono-centric urban patterns while the simulation was produced regardless of the location of the urban center. Experimental results show that using the WSF dataset, CityGAN generates precise urban patterns for Indian cities.

### Relationship between human settlement and transportation network of real cities

**Figure 7:**
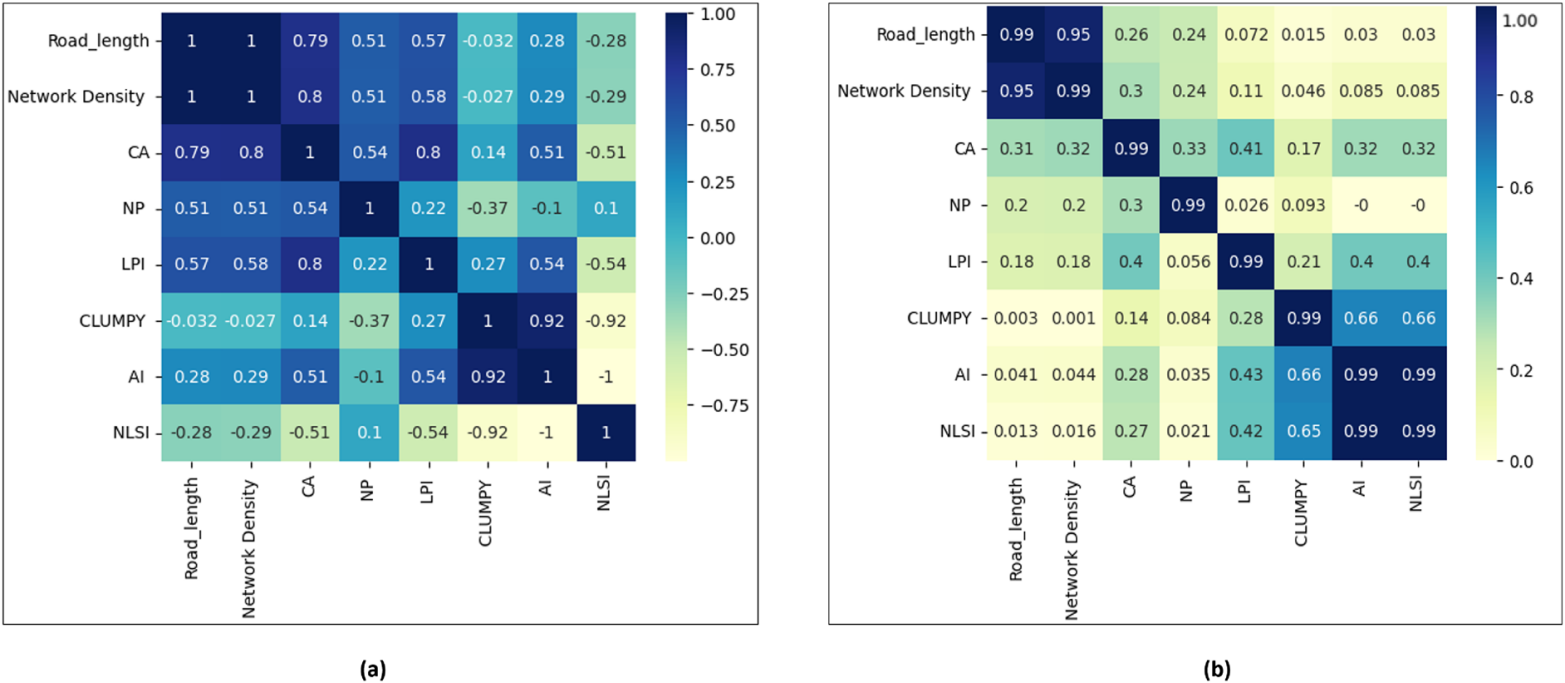
Heat map of the correlation between transportation indices and human settlement indices, the (**a**) PCC, and (**b**) CCC based on the input data. Heat maps were generated using the Python programming language (URL: https://www.python.org/).

HSIs and TI are computed for the selected cities (workflow is illustrated in Fig. 3b,c). The outcomes of the analysis of set human settlement using selected indices are displayed in Table 1. Table 2 provides examples of the calculation of the transportation index (network density) and Table 3 shows descriptive statistics of road length. Table 3 represents the analysis and summary of a numerical value to describe and understand the characteristics of road length of all the selected 503 Indian cities. The result shows the spatial distributions of road patterns vary among cities. Once the metrics are derived, correlation coefficients (CC) are calculated to determine the relationship between the human settlement indices (CA, NP, CLUMPY, LPI, AI, and NLSI) and the transportation index (RL, ND). The heat maps of the correlations between transportation indices and human settlement indices are illustrated in Fig. 7.

**Table 1:** Human settlement indices of small and medium-sized towns/cities in India calculated for the year 2019.

Grid number	City name	CA	NP	LPI	CLUMPY	AI	NLSI
G1	New Delhi	4054.11	5299	3.782	0.680	79.607	0.203
G2	Belgaum	1534.05	5999	0.234	0.637	68.421	0.315
G3	Gulbarga	2377.14	5722	0.844	0.652	72.184	0.278
G4	Jamnagar	2141.93	3003	1.857	0.733	78.272	0.217
G5	Dhulia	1608.7	4145	0.629	0.692	73.565	0.264

**Table 2:** City number (n), the total length of the road network (RL), city area (A) and network density (ND).

SI No	City name	n	RL (km)	A (sq. km)	ND
1	New Delhi	G1	1898.33	110.25	17.218
2	Belgaum	G2	1313.71	110.25	11.916
3	Gulbarga	G3	1714.51	110.25	15.551
4	Jamnagar	G4	1454.75	110.25	13.195
5	Dhulia	G5	1164.13	110.25	10.559

**Table 3:** Descriptive statistics of transportation metrics (namely road length).

Transportation metrics	Min	Max	Mean	Std. deviation
RL	15.7	2791.51	434.514	392.389

Figure 7a demonstrates the value matrix of Pearson’s correlation coefficient (PCC) as a measure of a linear relationship (ranges between $-1$ and 1). Its value reflects the strength of the link between metrics. Positive numbers demonstrate the beneficial influence of variables on each other, and negative values represent the negative connection between variables. Here, the correlation index is displayed by the color intensity as well. The correlation increases as the color bar rises, a light yellow color denotes a lower correlation. As shown in Fig. 7a, the PCC values of transportation indices RL and ND are highly related to each other; therefore we choose ND as the response variable of TI. Settlement metrics such as CLUMPY and NLSI have the minimum correlation with transportation metrics (light blue color), while CA demonstrates the maximum value of correlation coefficient with transportation indices. The correlation coefficients of RL and ND with CA are 0.7 and 0.80, respectively. According to PCC, the highest correlation exists between CA and RL, CA and ND. Hence, CA is an inevitable variable in our regression model. Nonlinear relationships between HSIs and TIs are explored using Chatterjee correlation coefficients (CCC) and reported in Fig. 7b. In the case of CCC, values reflect that most variables have positive correlations and CA has the highest rank followed by NP and LPI.

### Prediction of transportation index

A supervised machine learning approach (Kernel Ridge Regression) was implemented to predict road network density for given settlement patterns (refer to Fig. 3d). We compare the performance of various other supervised regression models such as support vector regression (SVR), decision tree (DT), gradient boosting (GB), multilayer perceptron regression (MLP), XGboost (XGB), linear regression (LR), random forest (RF) and simple ridge regression (RR) to select the best-fit model. To validate the models, four statistical scores: mean squared error (MSE), mean absolute error (MAE), R-squared, and adjusted R-squared were used. The human settlement indices (HSIs) were derived from settlement maps. Their corresponding network density (TI) values from OSM maps were utilized in the development of the KRR model. The model was implemented to predict road network density based on given settlement patterns. For training and assessing the prediction model, the original dataset consisting of 503 real city statistics (HSIs and TIs) was divided into two subsets (80% for training and 20% for testing). To validate the RidgeGAN model, we utilized the OSM dataset, and the accuracy of the KRR model predictions was then validated using the subset (20%) of the network density dataset derived from this OSM dataset. The results of our model and comparison with other models are summarized in Table 4. Experimental result shows that KRR outperforms all other eight state-of-the-art regression models, in terms of the highest $R^2$ and adjusted $R^2$ and lowest error metrics (MSE and MAE values). Because of the ability to handle nonlinearity and multicollinearity difficulties within datasets, the KRR regression model performs best. Validation metrics indicate that our model is good at predicting network density for urban patterns. This implies that the supervised KRR model can be applied to predict TI for newly generated cities by CityGAN.

**Table 4:** Performance metrics for the test set of the real dataset.

Accuracy metrics	SVR	DT	GB	MLP	XGB	LR	RF	RR	KRR
MSE	6.439	5.792	5.265	4.505	4.448	4.343	4.133	3.998	**3.661**
MAE	1.832	1.716	1.688	1.582	1.535	1.576	1.475	1.511	**1.397**
$R^2$ Score	0.490	0.541	0.583	0.643	0.648	0.656	0.673	0.683	**0.710**
Adj $R^2$ Score	0.463	0.517	0.561	0.624	0.629	0.638	0.655	0.667	**0.695**

The best model’s values are highlighted in bold.

## Discussion

India is now the most populous country in the world^12^ with more than 30% of the population residing in the urban area. Government of India recently came up with a scheme called IDSMT^15^ to improve urban planning and road networks for the development of small and medium-sized cities (population size up to 500 k). The Ministry of Urban Development offers financial and technical assistance to local bodies in developing their region’s infrastructure and basic services to promote inclusive growth and balanced regional development. One of the objectives of this scheme is to address the transportation infrastructural deficits and service shortfalls of the eligible cities. However, the Central Government finds difficulty in making a correct decision to allocate funds for transportation infrastructure development.

This study proposes a hybrid model to predict road network density for small and medium-sized settlement patterns in India. We used the publicly available WSF datasets and CityGAN model to build an unsupervised model to simulate realistic Indian urban patterns. The average radial profile was used to compare real against simulated cities to validate the performance of CityGAN. Also, we used the K-means technique to cluster the radial profiles with the optimal number of clusters equal to 10 for both actual and synthetic scenes using a straightforward fraction of sum-of-squares reasoning. Landscape structures of these generated cities were measured in terms of human settlement indices using spatial landscape metrics. Then, various supervised machine learning models were implemented for predicting transportation indices from human settlement indices based on real city datasets. All the regression models were compared based on error measurement metrics. The performance of the KRR model outperformed the benchmark regression methods. The transportation index estimated from the KRR model is compared with actual test data (from real cities). KRR has comparatively less MAE of 1.39 and a higher $R^2$ value of 0.71. Thus, the proposed hybrid model can be used to predict the transportation index in terms of road network density for CityGAN-generated towns and cities. Our proposal can be treated as a versatile decision-support system for sustainable planning and development of new small and medium-sized towns and cities in terms of transportation infrastructure.

However, the effectiveness of the proposed model may vary depending on factors such as the complexity of the urban landscape, the diversity of the training dataset, and its availability. Larger cities tend to have more diverse and intricate urban patterns, which might pose challenges in building the CityGAN model. Some potential limitations include having substantial variations in the urban landscape between large cities and small and medium cities. The model might not perform as well when applied to areas with more diverse characteristics. Hence, generating realistic large Indian urban landscapes using CityGAN requires large and diverse datasets. The availability of the number of data points will be limited for large cities as compared to small and medium-sized cities of India. The proposed method is useful for establishing a relationship between coverage measures (network density) of transportation indices with HSIs, but the current work doesn’t consider any connectivity measure. As a future scope of work, making interconnections between connectivity measures with HSIs will be worth exploring.

## Methods

In this section, we introduce the components of the model used in the hybrid framework are demonstrated. First, we discuss various correlation measures that are used in this study. Further, our proposed two-step pipeline approach utilizes the popularly used GAN model (CityGAN) in this case and the KRR model, a nonlinear shrinkage method for regression modeling. After this, we go over the suggested RidgeGAN method. Figure 3 illustrates the connection between the two-fold objectives of our research. Existing methods in this field only talked about the generation of urban patterns (for e.g., CityGAN and MetroGAN) using urban footprint images but not for Indian cities. Also, to our best knowledge, no empirical study has yet been reported regarding the connection between the transportation index and human settlement indices. Our study fills this gap in the literature with the two-fold objectives. Such research is of considerable significance in the realm of urban planning particularly in rapidly developing nations like India and also for a better understanding of urban transport systems and human settlements. Therefore, the primary goal of our research consists of the following two main objectives: (1) generate realistic urban patterns to capture the great diversity of urban forms across small and medium-sized Indian cities by using CityGAN; (2) disentangling urban human settlement indices in the population dynamics and establish a nonlinear association between road network density and human settlement indices. We utilize a uniform and standardized two-step approach to preprocess open datasets from diverse built environment domains (WSF 2019 and OSM) and to implement the integrated model.

### Correlation analysis

Correlation coefficients (CC) are popular statistical measures used to determine the strength of a relationship between two or more variables (can be numerical or categorical). Pearson’s correlation coefficient (PCC) is the most commonly used classical method of measuring linear associations, and its ease of use is advantageous^61^. However, their efficiency may be limited when dealing with non-normal, noisy, closed, or open data (even after applying log ratios to the data). Chatterjee Correlation Coefficient (CCC)^62^, a recently developed method based on cross-correlation between ranked increments, is a reliable alternative to traditional correlation methods. CCC can deal with data that contains outliers or has non-normal distributions and it does not make any assumptions about the data distribution^61^. A Python implementation of CCC can be done using the “TripleCpy” package in Python. We can define CCC mathematically as follows: given a pair of random variables (*X*, *Y*) and suppose the realizations $X_i$’s and $Y_i$’s have no tie. A rearrangement of the pairs as $\left( X_{(1)}, Y_{(1)}\right) , \ldots ,\left( X_{(n)}, Y_{(n)}\right)$ is done such that $X_{(1)} \le \cdots \le X_{(n)}$. Let $r_i$ be the rank of $Y_{(i)}$ then CCC is defined using the formula:

$$\begin{aligned} \xi _n(X, Y):=1-\frac{3 \sum _{i=1}^{n-1}\left| r_{i+1}-r_i\right| }{n^2-1}, \end{aligned}$$

where *n* is the number of observations. Once the relationships are assessed between TI and HSIs, selecting the “best” regression model is easier so that the model explains the variability in the response variables with possibly lower prediction error. However, the ridge regression (RR) method can directly handle multicollinearity structures in the data along with the instability of least square estimators^63^. A more effective nonlinear regularized regression technique in machine learning is kernel ridge regression (KRR). The creation of the ridge regression method addresses some of the shortcomings of the least square method (over-fitting and multicollinearity)^64^. One of their advantages is that the kernel implementation allows to handle nonlinearity of the data^65–67^.

### CityGAN: generative adversarial networks for modeling urban patterns

GAN is a powerful unsupervised deep learning model that learns representations of input data to fit high dimensional complex distributions^18^. GANs are revolutionary in that they can produce very high-quality (i.e., extremely realistic) samples compared to predecessor models at similar computational costs. In general, GAN is a system that consists of two neural networks competing against each other in a zero-sum game context^68^. The two neural network architectures are a generator (*G*) and a discriminator (*D*) and they can generate new data that conforms to learned patterns through both generative and adversarial processes. GANs demonstrate promising performance in modeling complex geospatial data having spatial dependence^22^. Whilst the core application of GANs has been computer vision and image processing^18^; however, their use in geoscience has provided urban planners with novel ways of generating “new” samples that can easily outperform state-of-the-art geostatistical tools. In this study, we deploy CityGAN^23^ to learn the urban patterns from real settlement images and generate hyper-realistic urban settlement images. *G* and *D* are both deep convolutional neural networks with weight vectors $\theta _G$ and $\theta _D$. Back-propagation is used to learn these weights by alternatively reducing the following loss functions

3 $$\begin{aligned}{} & {} \theta _D: \mathscr {L}_D=E_{h \sim p_h}[\log D(H)]+E_{z \sim p_z}[\log (1-D(G(z)))] \end{aligned}$$

4 $$\begin{aligned}{} & {} \theta _G: \mathscr {L}_G = E_{z \sim p_z}[\log (1-D(G(z)))] \end{aligned}$$

Here, the generator is made up of numerous convolutional blocks, including inverse-convolutional, batch normalization, and rectified linear unit (ReLU) layers, and ends with a hyperbolic tangent layer (which applies tanh ($\cdot$) nonlinearity to each element of the produced map). Recent modifications of GANs have allowed the performing of conditional generation as domain transformation. In the GAN training phase, it is worth noting that the generator network is usually able to create realistic samples whereas the discriminator is an auxiliary network that gets discarded after training. Once GAN is trained, the CityGAN^23^ can be used to generate new synthetic urban images that can be used for a variety of applications such as urban planning, disaster response, and simulations. Iteratively optimizing the *G* and *D* networks is part of the training process. The generator network attempts to deceive the discriminator by generating images that resemble real urban images, whereas the discriminator network attempts to correctly classify whether an image is real or fake. The networks are updated based on classification and generation errors until the generator produces images that are indistinguishable from real-world human settlement scenes. $H_{fake}$ is implicitly sampled from the data distribution that the generator tries to imitate when G is at its optimum. However, the GAN-generated images may not be representative of all possible urbanization patterns because they are based on the training dataset and the GAN architecture used. As a result, before using GAN-generated images for any practical application, it is critical to carefully evaluate them and compare them to real-world urban areas.

### Kernel ridge regression (KRR)

Regression modeling is a fundamental area of machine learning where the target variable is quantitative (real numbers) in nature. The most classical approach is linear regression using the ordinary least square method. However, it has salient disadvantages, e.g., overfitting and multicollinearity which can be addressed via ridge regression. Ridge regression “shrinks” the least square coefficients using regularization parameter via minimizing the objective function given below:

5 $$\begin{aligned} \text {Ridge}(\beta ) = \frac{1}{2}(Y-X\beta )^{T}(Y-X\beta ) +\frac{\lambda }{2}\beta ^{T}\beta , \end{aligned}$$

where $X \in R^{N \times D}$ is the feature matrix with *N* being the number of training samples, *D* is the number of features $Y\in R^1$ is the real-valued target vector, $\beta$ is the regression coefficients, and $\lambda \ge 0$ is the regularizer that helps in dealing multicollinearity problem. However, the Ridge regression model still has troubles when dealing with nonlinear data data^69^. A more general framework can be achieved by using a nonlinear mapping function $\phi (\cdot )$ that maps low-dimension features to high dimension (helps in learning nonlinear patterns). Now, a kernel function in the form of the dot product is used to avoid the cause of dimensionality of the nonlinear transformation. Mathematically, Kernel between two points, say $x_m$ and $x_n$ is given by

6 $$\begin{aligned} K(x_m,x_n)= \phi (x_m)^T\phi (x_n), \end{aligned}$$

which satisfies Mercer’s condition^70^. The major impact of Kernel is ridge regression which allows the identification of nonlinear functional relationships between one variable with remaining features. In this study, we tuned radial basis kernel function (RBF)^67^ which is defined by:

7 $$\begin{aligned} K(x_m,x_n)= e^{-\gamma \Vert x_m - x_n\Vert ^2}, \end{aligned}$$

where $\gamma$ is the width of the kernel. Predictions in KRR model for a new test input $x_{*}$ is given by,

8 $$\begin{aligned} \beta ^{T}\phi (x_{*}) = \sum _{n=1}^{N} (XX^{T}+\lambda I_{N})^{-1}Y K(x_n,x_*). \end{aligned}$$

The regularization parameter (Alpha) is set as 0.02; it controls the trade-off between fitting the training data and prevents overfitting. The input features are a wide range of human settlement indices related to network density considered. We selected six settlement indices such as total class area (CA), number of patches (NP), largest patch index (LPI), clumpiness index (CLUMPY), aggregation index (AI), and normalized landscape shape index (NLSI) to represent the characteristics of human settlements. The target variable is network density. We use KRR to establish the relationship between HSIs and TI as shown in Fig. 3d.

### Hybrid model: RidgeGAN

RidgeGAN is a hybrid approach based on unsupervised CityGAN and supervised KRR models. KRR^67^ has a built-in mechanism to perform nonlinear regularization analysis in the presence of multicollinearity. CityGAN^23^ became popular to generate fake city images (a.k.a possible future cities) that look like real cities from the visual inspection and are statistically significant via learning the urban morphology. After implementation, we evaluated the performance of CityGAN, by comparing the real and simulated cities using the most widely used spatial summary statistics in an urban analysis called average radial profile and peak search algorithm. Each city is represented as a $10.5\times 10.5$ km image covering the urban center and surrounding regions. However, the quantifying transportation index has a vital role in the development of sustainable city planning and management. Here, we built a supervised KRR model to predict the transportation index by learning the relationship between urban patterns and the road transportation index. The KRR prediction model is integrated with the CityGAN model to predict the transportation index of newly generated cities using CityGAN. To build our hybrid model, we mainly use two models: an unsupervised learning model for generating urban patterns and a supervised learning model to predict the transportation index. To sum up, the workflow of the proposed RidgeGAN is detailed as follows (also see Fig. 3 for a schematic workflow):

- First, we apply CityGAN, an unsupervised learning model to generate small and medium-sized Indian cities using the available urban maps.
- Landscape structures of real and generated cities are measured in terms of Human Settlement Indices (HSIs) using spatial landscape metrics.
- We assess the relations between two important features of urban forms (human settlement and transportation system) and build a KRR model to predict the transportation index, namely network density.
- The proposed hybrid model framework can predict the road network density on a given urban pattern for the urban maps generated in the first step.

### Key contributions of the RidgeGAN model

Currently available literature in the field of “Modelling Urbanization Patterns and Simulating Urban Morphology”^23,24,44^ focuses on using GANs (Generative Adversarial Networks). For example, Albert et al.^44^ proposed the use of unconstrained GANs and later showcased the usage of physics-contained GAN to model urbanization patterns and predict land use. Zhang et al.^24^ have focussed on simulating urban morphology with progressive growing structures while imposing physical geography constraints with geographical loss.

Despite existing research using GANs to study the spatial effects of urban systems gaining attention, their applications to urban science are still unexplored. As such, the available literature does not discuss the mapping of urban attributes to urban morphology. On one hand, different important aspects of urban forms are very well captured through spatial metrics such as the number of patches, Clumpiness/Aggregation index, Largest patch, Class area, etc. On the other hand, connectivity and coverage measures have been widely used as topological/transportation indices (for example, urban road networks) that define mobility and transportation within urban cities. To the best of our knowledge, no empirical studies have explored the inherent relationship/association between spatial metrics (that depict human settlement patterns) and transportation indices.

Therefore, the current study is an attempt to address the following two objectives: (1) to use GAN for modeling urban morphology while simulating the spatial structure of cities. GAN is used here to study the spatial effects of geographical systems. The authors understand that there are studies that address urban growth patterns from human mobility behavior^71^ to models that are based on complexity science such as Cellular Automata^72^, etc. These approaches model city data from a bottom-up perspective and capture key growth mechanisms but the generated form is not similar to the real-world situation at a country-to-continent and global scale because they tend to ignore some complex high dimensional features of urban morphology^72^. Therefore, the choice of using GAN is based on promising results owing to its ability to approximate complicated high-dimensional probability distributions^73^. We hope to further realize its potential in urban science. (2) To develop a spatial relationship model between derived spatial metrics and topological indices from 500 real city images using Kernel Ridge Regression. Further, the developed model was used here to predict the topological index (Network Density) for the simulated images that have similar urban forms and patterns as that of real cities obtained from a spatially explicit model of urban patterns based on GANs that is characterized by very limited spatial information.

Therefore, this study is a value addition and contribution to realizing the relationship between spatial metrics and transportation indices within real and simulated Indian cities (as an example) with the aid of the urban universe. Prior literature on urban analysis almost entirely focussed on cities in developed countries due to rich data availability. However, projections reveal that most of the urbanization is going to happen in developing countries in Asia and Africa in the absence of data-informed regulatory policies. In many such countries like India, socioeconomic, demographic, and economic data are limited and unreliable. Together, the limitations of existing traditional urban models (discussed above) that need detailed GIS data layers and do not scale from country to global levels along with the urgent need for data-driven decisions for expanding cities in developing countries, motivated us to develop and build a hybrid predictive model (RidgeGAN) that were calibrated using a globally consistent data. Our proposal is not only able to capture the urban morphology of a large number of Indian smaller and medium-sized cities but also gives insight into the network density prediction (i.e., transportation index) through a set of spatial metrics (i.e., settlement indices). For this, we have utilized a uniform and standardized two-step approach to pre-processing open source datasets from diverse built environment domains (WSF2019 and OSM Data) to implement the integrated hybrid model. The study serves as proof of the application of a hybrid model for interpretability and usability in urban morphology prediction for Indian cities.

### Drawbacks, limitations, and future scope of the study

**Figure 8:**
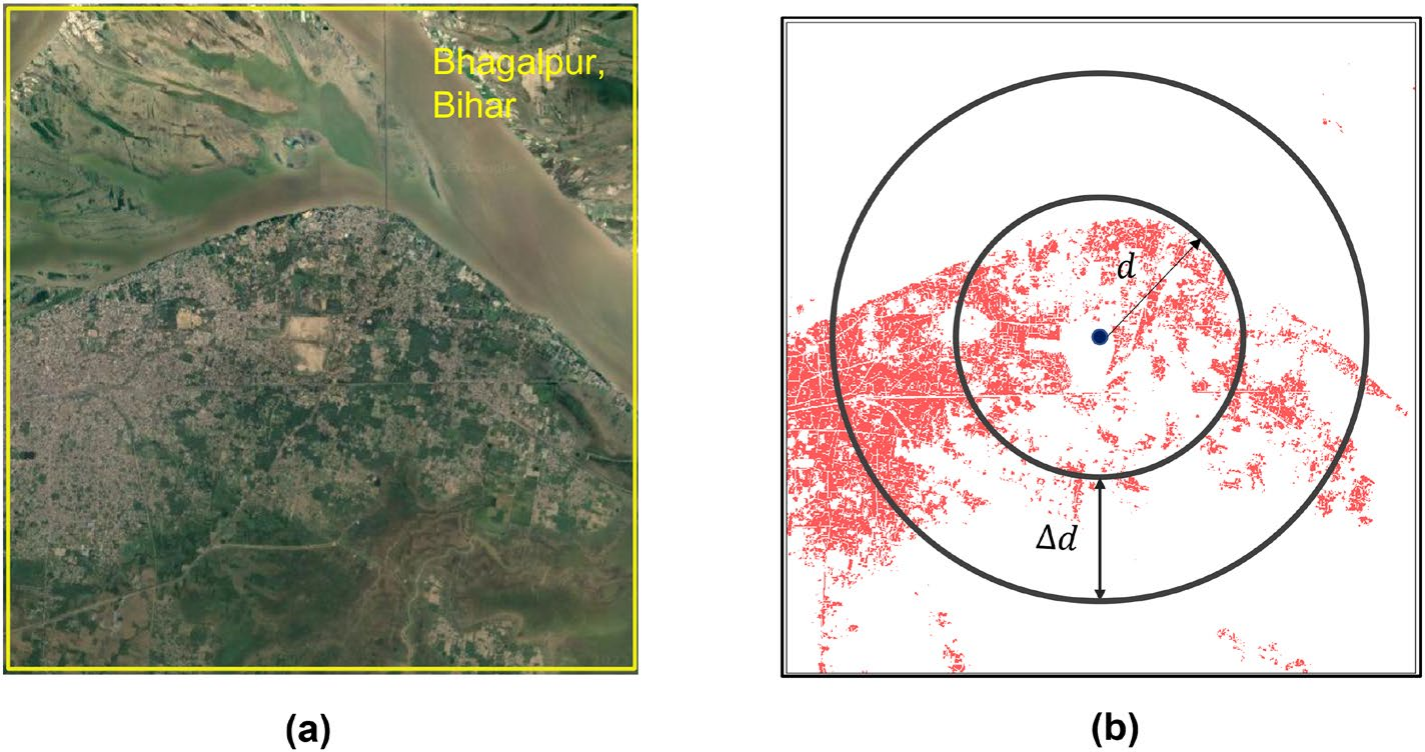
(**a**) Google satellite view of Bhagalpur city (randomly selected city to explain) in Bihar state. Source: Maps data: Google, ©2015 India (URL: https://www.google.com/maps); (**b**) human settlement map with the method of computing their average radial profiles.

As our proposed hybrid approach uses two key models: KRR and CityGAN, there are a few limitations of the proposal. In contrast to conventional deep learning architectures that employ convergence-based optimization of the objective function, generative models like GANs utilize a minimax loss function, trained iteratively to establish equilibrium between the generator and discriminator networks^73^. The absence of an objective loss function for GAN training restricts the ability of loss measurements to assess training progress or model performance. To address this challenge, a mix of qualitative and quantitative GAN evaluation approaches has been developed^74^. These evaluation measures particularly vary based on the quality and diversity of the generated synthetic data, as well as the potential applications of the generated data^75^. For our case, we have used ARP and peak search algorithm; however, they fail when it comes to time-varying urban patterns and enormous data for analysis (Fig. 8).

Another salient limitation of the proposed framework is as follows: although this study evaluates the relationship between HSIs and TI based on OSM data, this validation can not be performed on the simulated data for which the network density is not available. However, using the RidgeGAN model, urban planners can estimate (or make forecasts) the road network density of the simulated data (still evaluating their accuracies remains a future challenge). This study promises several future scopes for research:

1. Future study will focus on including more spatial layers in the input data such as historical and temporal urban data, Digital Elevation Model (DEM), Night Time Light Data such as from DMSP OLS^76^ and NPP VIIRS^77^ data as a proxy of economic activities, Water layer, etc. Incorporating constraints and conditions as part of the input to the model will also be explored.
2. It is acknowledged that the spatial statistics used in this study to validate the model output do not consider all of the richness in the spatial form. Future studies will develop a comprehensive suitable evaluation framework and metrics for assessing urban morphology for simulated cities at multiple aggregation scales. This would answer questions such as how far the simulated cities mimic the real cities in terms of latent features and morphology from pixel to macroscopic levels. Improvement of the existing model will be attempted to account for the latent space representations of the important characteristics of urban spatial maps.

## Data Availability

The datasets and the codes for implementing the methods are made available at https://github.com/Rahisha-Thottolil/RidgeGAN.
